# A Virtual Reality Exergame to Engage Adolescents in Physical Activity: Mixed Methods Study Describing the Formative Intervention Development Process

**DOI:** 10.2196/18161

**Published:** 2021-02-04

**Authors:** Nuša Farič, Lee Smith, Adrian Hon, Henry W W Potts, Katie Newby, Andrew Steptoe, Abi Fisher

**Affiliations:** 1 Department of Behavioural Science and Health University College London London United Kingdom; 2 Faculty of Science and Engineering Anglia Ruskin University Cambridge United Kingdom; 3 Six to Start London United Kingdom; 4 Institute of Health Informatics University College London London United Kingdom; 5 Department of Psychology and Sports Science University of Hertfordshire Hatfield United Kingdom

**Keywords:** adolescent, adult, exercise, leisure activities, obesity, sports, video games, mobile phone, virtual reality, motivation

## Abstract

**Background:**

Early adolescence (13-17 years) is a critical developmental stage for physical activity promotion. Virtual reality (VR) exergaming is a promising intervention strategy to engage adolescents in physical activity.

**Objective:**

The vEngage project aims to develop a physical activity intervention for adolescents using VR exergaming. Here, we describe the formative intervention development work and process of academic-industry collaboration.

**Methods:**

The formative development was guided by the Medical Research Council framework and included recruiting an adolescent user group to provide iterative feedback, a literature review, a quantitative survey of adolescents, qualitative interviews with adolescents and parents, inductive thematic analysis of public reviews of VR exergames, a quantitative survey and qualitative interviews with users of the augmented reality running app Zombies, Run!, and building and testing a prototype with our adolescent user group.

**Results:**

VR exergaming was appealing to adolescents and acceptable to parents. We identified behavior change techniques that users would engage with and features that should be incorporated into a VR exergame, including realistic body movements, accurate graphics, stepped levels of gameplay difficulty, new challenges, in-game rewards, multiplayer options, and the potential to link with real-world aspects such as physical activity trackers. We also identified some potential barriers to use, such as cost, perceived discomfort of VR headsets, and motion sickness concerns. A prototype game was developed and user-tested with generally positive feedback.

**Conclusions:**

This is the first attempt to develop a VR exergame designed to engage adolescents in physical activity that has been developed within a public health intervention development framework. Our formative work suggests that this is a very promising avenue. The benefit of the design process was the collaborative parallel work between academics and game designers and the involvement of the target population in the game (intervention) design from the outset. Developing the game within an intervention framework allowed us to consider factors, such as parental support, that would be important for future implementation. This study also serves as a call to action for potential collaborators who may wish to join this endeavor for future phases and an example of how academic-industry collaboration can be successful and beneficial.

## Introduction

### Background

The benefits of performing sufficient physical activity (PA) are well established and include prevention of noncommunicable disease and better mental health [1,2]. Early adolescence (12-17 years) [3] is particularly important because there is a substantial age-related decline in activity levels from childhood into adolescence [4], and active adolescents are more likely to become active adults [5]. In addition, sustained moderate-to-vigorous PA (MVPA) in adolescence is positively associated with multiple markers of metabolic health, such as blood pressure levels, insulin, C-reactive protein, lipoprotein cholesterol, and triglycerides among others [6]. There is also some evidence that depression and anxiety rates are lower in more active adolescents [7], and PA reduces depressive symptoms in this group [8]. However, most of the adolescents are insufficiently active. Engaging adolescent girls in PA is particularly important because the age-related decline is greater for girls [9]. Objectively measured data from the Health Survey for England in 2016 showed that less than 15% of UK adolescents met the government’s PA recommendations of at least 60 min MVPA per day [10]. Similarly, the United States Department of Health and Human Services estimates that over 80% of American adolescents do not meet guidelines [11], and low PA levels are observed globally [12,13]. Leisure-time PA throughout adolescence is increasingly replaced by sedentary behaviors such as screen time [14], highlighting screen time as a potential intervention target.

### The Challenge of Engaging Adolescents in PA

Although it is clear that an intervention is required, how to change adolescent PA behavior at a population level remains unknown. A 2012 meta-analysis of 30 randomized controlled trials of PA interventions in children and adolescents up to 16 years of age found that interventions had negligible to small effects on accelerometer measured total activity levels and small effects of MVPA, equivalent to approximately 4 additional minutes per day. Only two of these interventions specifically targeted adolescents [15]. A 2019 meta-analysis of 17 school-based interventions, including children and adolescents, found no increase in objectively measured daily MVPA in the intervention group compared with control groups [16]. These authors suggested that interventions were failing at the implementation stage, and the paper was a call to action for a careful description of the intervention development and process evaluation [16]. A multimodal approach may be required to target school or home environments, policies, and parents [17,18]. However, multicomponent interventions are labor-intensive, and it remains unclear how feasible they are for wide-scale implementation. Digital interventions have been proposed as a solution, but their efficacy for promoting PA in adolescents is not clear. A systematic review identified 17 digital interventions for adolescents designed to promote PA and diet, but studies were generally small and had mixed findings [19]. In addition, the majority were web-based, which may not reflect current volitional adolescent technology behavior [19].

### Gaming to Engage Adolescents in PA

Some industries have been very successful in engaging adolescents, particularly the gaming industry. Gaming is a recreational activity regularly performed by over 90% of adolescents, popular across socioeconomic groups and with girls and boys [20]. Gaming was recently highlighted as a promising avenue for health promotion [21]. Games requiring bodily movement—exergames—can encourage PA. A meta-analysis of 35 trials in children and adolescents found that previous generation exergames such as Wii Fit and Dance Dance Revolution had a similar physiological benefit to *active* controls (running or field-based PA) [22]. Exergames also enhanced self-efficacy, liking or enjoyment, and intrinsic motivation for PA [22]. In a 3-arm randomized controlled trial of participants aged between 15 and 19 years with obesity, those encouraged to play Wii Fit for 40-60 min per day cooperatively (working with a peer to expend calories) lost significantly more weight and had increased self-efficacy compared with competitive (competing against each other) or control conditions (regular daily activities) [23]. In another small trial by the same researchers, the percentage of body fat was reduced in girls who adhered to a dance exergame [24]. Although most exergames were not designed in a research context, McBain et al [25] developed a high-intensity training (HIT) exergame for adult males in deprived communities and found that the game delivered sustained progressive HIT training over a 6-week pilot [25]. A mobile app-based game called *Pokémon Go*, which has activity as a byproduct of the gaming and fun element, has had huge commercial success—with over 800 million downloads—and resulted in short-term increases in users’ step count [26]. A longitudinal survey found that exergaming may be effective in engaging adolescent girls in sustained PA and that gender and motivation may be particularly important to consider when designing exergames [27].

The gaming industry has been commercially successful in building engaging products, but these were not developed as public health interventions or evaluated using traditional academic approaches. Collaboration between sectors offers a solution but can pose challenges, with industry and academia having different skills, goals, timeframes, epistemic cultures, and definitions of success [28]. The challenges of cross-sectoral collaboration between industry and academia and health services have long been recognized as barriers to innovation adoption in health care [20]. There are also differences within academia between researchers from a health background and those from a computing background. Blandford et al [29] note the contrast between the former having a focus on summative evaluation and the latter focusing on formative evaluation, as well as the differences in epistemology and reporting culture [29]. Academic computer science research is closer to software industry practice in a number of ways, such as the nature of filling the gaps in research (summative vs formative reviews), the accepted evidence, and the way research is reported [29].

### Potential of Virtual Reality

As part of a previous small industry-partnered innovation grant designed to explore the potential of virtual reality (VR) to educate young people about the benefits of PA, *Innerselfie* [30], we conducted technology workshops with adolescents aged between 13 and 17 years allowing them to try top range virtual and augmented reality (AR) and observed how engaging they found immersive VR. VR has the potential to enhance the impact of an exergaming experience, as VR creates presence and allows the user to be fully immersed in the virtual environment [31]. VR headsets are increasingly portable, and wireless headsets and tracking allow for whole-body movement. Statista reported projected worldwide shipments of VR headsets to reach 15 million in 2022 [32]. There is early evidence from laboratory studies in adults that immersive VR exergaming is more engaging than standard exercise. For example, in 88 university staff and students, heart rate was higher in VR versus standard exercise conditions, with lower fatigue ratings and higher enjoyment in VR [33]. A pre-post study including 9 children found that playing an older generation VR exergame, *Astrojumper*, for 15 min enhanced self-reported motivation to exercise, but this study had no follow-up to test whether behavior changed [34]. However, to the best of our knowledge, when we began this work, there were no VR exergaming interventions designed to promote PA in adolescents and none where substantial formative development work had been conducted to inform content.

For the work presented in this paper, we followed the Medical Research Council (MRC) framework for developing and evaluating complex interventions published in 2000 and updated it in 2019 [35]. MRC guidance consists of 5 steps, (1) developing, (2) piloting, (3) evaluation, (4) reporting, and (5) implementation, specific to epidemiology, public health, and even social policy [35]. In line with the MRC framework of developing interventions and other coherent approaches to intervention development [35], substantial formative development work is recommended to understand the target behavior and end users’ views. Although the interventions will likely need to target multiple social and environmental determinants of PA to achieve sustained PA change [36], we hypothesized that VR exergaming could be a platform to engage and motivate adolescents to be physically active and could ultimately form the core of a multidimensional intervention. However, it was recognized that to make a high-quality, appealing VR exergame that adolescents would want to play, industry experts would have to lead the development. Here, we describe the steps involved in the early intervention development process of a true industry-academic partnership aimed at designing a VR exergaming intervention for adolescents.

### Objectives

The overarching objective of the vEngage project is to develop and test a PA intervention for younger adolescents involving VR exergaming. The purpose of this paper is to describe the process of the academic-industry partnership, formative development studies that fed into game development, and user testing of the prototype VR game. Future phases (subject to funding) will involve developing the game to full specification and identifying additional components (including *real-world* activity partners) to encourage sustained behavior change.

Funding was awarded by the UK MRC Public Health Intervention Development scheme to conduct the formative stages of intervention development outlined in Table 1. The project was a true academic-industry partnership whereby the industry partner Six to Start (led by AH) was a coinvestigator on the grant and involved since the project’s inception; the grant gave structure to the collaboration. The collaboration was only possible because of the prior *problematization* [37] based on Six to Start’s previous experience with the National Health Service, interest in the promotion of PA, and establishment of relationships across the research team (AH had been on the Innerselfie steering committee as an industry expert).

The academic team was led by AF, a behavioral scientist with a background in promoting PA for the prevention and treatment of chronic diseases. Other academic partners have expertise in sports science and epidemiology (LS), health informatics and statistics (HP), psychology and epidemiology (AS), risk perception and health behavior (KN), and psychology (NF). The industry partners (led by AH) are Six to Start [38], commercial game designers who developed the world’s most successful mobile app AR narrative (audio)–based exergame Zombies, Run!

**Table 1:** Steps in phase I vEngage intervention development work.

Academic team			Update meeting	Industry team
**Completed steps: phase I**				
	**MRC^a^ step 1: developing**			
		1. Literature review to identify determinants of PA^b^ and mechanisms of change	X^c^	Scoping and finalizing suitable technologies
	**MRC step 2: piloting**			
		2. Recruit user group	X	Set up development environment and toolchain
		3. Pilot quantitative survey with 695 adolescents	X	Preconception and technology feasibility
		4. Qualitative interviews with adolescents and parents	X	Preconception and technology feasibility
		5. Thematic analysis of public reviews of VR^d^ exergames	X	Preconception
		6. Survey of Zombies, Run! users with qualitative interviews	X	Develop and iterate prototype game using real hardware and exercise integration
**Future steps: phase II**				
	MRC step 3: evaluation and MRC step 4: reporting			
	**MRC step 5: implementation**			
		7. User/steering committee testing	X	Prototype released to the research team
		8. Pre-post study	—^e^	Not applicable for the industry team
		9. Outline components for community PA link up and parental support	—	Outline necessary iterations to prototype game and possible link up technologies to integrate into game (eg, smartphone tracking)

^a^MRC: Medical Research Council.

^b^PA: physical activity.

^c^Steps completed by both teams.

^d^VR: virtual reality.

^e^Data not available.

## Methods

### Process for the Academic-Industry Collaboration

An important aspect of our intervention development was managing the academic-industry collaboration. It was essential to establish a process that could balance the need for formative development work using traditional research processes with the speed of working of game designers in a fast-moving technology-based industry within the period of grant funding for phase I (12 months). Ideally, formative work would have been conducted before game development, but this was not feasible within the funding period, so work had to be conducted in parallel, with ongoing dialog and regular meetings involving presentations of research findings (academic team) and game demonstrations (industry partners). Key steps and timings of meetings are shown in Table 1. The industry partner was always aware of the need for flexibility to be built into the game design in case new evidence arose from our work or the work of other researchers.

### Literature Review: Identifying Theoretical and Behavioral Constructs

A key *first step* in the research process was to identify which determinants may influence younger adolescents’ PA and therefore which theory may inform the intervention. NF extensively reviewed the literature and identified potentially modifiable targets (summarized in Table 2). The determinants identified most closely aligned with the Self-Determination Theory (SDT) of motivation [39], and it has been posited that the appeal of gaming may be grounded in their ability to satisfy basic psychological needs for competence, autonomy, and relatedness, core constructs of SDT [40]. The identified determinants and SDT were presented to the industry partners in our first formal meeting; then, the group discussed which would be feasible to target using the exergame component. As highlighted in the introduction, there is meta-analytic evidence that older generation exergaming can enhance PA self-efficacy, intrinsic motivation, and enjoyment in children and adolescents [22,41]. A pre-post VR exergame study in 9 children suggested VR exergaming may enhance motivation and enjoyment of PA [34]. Therefore, it was determined that a VR exergame could target PA self-efficacy, motivation, and identity by allowing young people to experience movement in an immersive and fun environment (Table 2). Determining the wider intervention components (Table 2) is a key part of phase II (future work, subject to funding).

**Table 2:** Behavioral determinants of adolescent physical activity from literature reviews and hypothesized method of inclusion in the intervention.

Potentially modifiable targets and description		Proposed intervention components	Proposed BCTs^a^ [42]
**VR^b^ exergame^c^**			
	Self-efficacy: individuals own beliefs about their capability to carry out a task	Game features that encourage feedback on performance, small in-game rewards, and multiplayer elements for social support/feedback on the performance of others	Feedback on performance Goal setting Rewards (in-game) Social support Time management
	Motivation: particularly intrinsic motivation driven by internal reward	Delivery of PA^d^ using a platform that is highly appealing to target users, perform PA in a fun and visually appealing immersive environment, and specific game targets to access next levels	Goal setting Self-reward Incentive
	PA identity: individuals view themselves as someone who is physically active (or not)	PA as a byproduct of fun and enjoyment, immersion as a distraction from negative physiological effects of exertion, and shifting negative perceptions of PA	Framing and reframing Identity associated with changed behavior
**Wider intervention^e^**			
	Parental support: identify ways to reduce concerns about gaming and garner parental support for intervention	Design a nonviolent game; Displace sedentary with active screen time	Social support Monitoring of behavior by others without feedback (awareness) Time management
	Community/external PA opportunities: link up with PA partners that appeal to target users	User group suggestions were web-based influencers and users of trampolines, skate parks, or climbing walls	Restructuring the physical and social environment Behavioral practice or rehearsal
	Link up with wearables and trackers: synchronize game with wearables or PA trackers to feed into the game	Most common or preferred by target group smartphone app	Self-monitoring of behavior Feedback on behavior
	Habits	Encourage game play at the same time and the same context to develop a PA habit; Displacing sedentary behaviors with active gaming	Habit formation Planning

^a^BCT: behavior change technique.

^b^VR: virtual reality.

^c^Focus of the current phase (although some aspects like multiplayer could not be built into the prototype).

^d^PA: physical activity.

^e^Future work, subject to funding.

### Behavior Change Techniques

The intervention required specific behavioral targets. It was not entirely possible to determine these from adolescent literature, since (as highlighted in the introduction) there is a lack of effective interventions to draw from. However, retrospective coding of more than 200 trials of PA interventions in more than 12,000 participants using behavior change techniques (BCTs) [43] found that BCT taxonomies such as goal setting, self-monitoring, and feedback on behavior were consistently associated with successful PA change [42,44]. The aforementioned review of digital interventions for adolescents also suggested that these BCTs may be particularly important [45]. However, part of the work within this grant (user group visits and quantitative survey with adolescents) aimed to determine which BCTs they would engage with in a digital intervention. Table 2 includes the proposed BCTs that could be linked to specific elements of the intervention.

### Establishing and Working With a User Group

A key part of our formative work was establishing a group of adolescent users to obtain iterative feedback on ideas, help us design questions for empirical research, and try technologies. It was important to ensure representation from girls and boys. A total of 36 participants aged 13-16 years (19 boys and 17 girls) from 3 London schools were recruited. The user group was separated from the research participants. A number of visits to schools were conducted to (1) discuss initial ideas to shape the grant application, (2) discuss experiences of gaming and VR and rank BCTs in order of preference, and (3) try our prototype game and consider what the wider intervention might contain.

### Quantitative Survey With Adolescents

A cross-sectional quantitative survey of adolescents was conducted through 2 London-based schools and web-based platforms (girls’ schools were recruited in the hope of understanding more about the views of girls, but the web-based version was open to anyone aged 13-24 years). The survey aimed to understand adolescent PA behavior, beliefs about and desire to change PA, and identify which BCTs they would like to see in a digital intervention. The survey also asked adolescents to report which platforms they used or preferred to help understand which companion technologies might accompany a VR exergame (to allow self-monitoring). Full analyses are in progress, but the summary findings using descriptive statistics (provided to the industry partner to incorporate into the game) are described in this paper.

### Qualitative Interviews With Adolescents and Parents

Semistructured interviews were conducted with target users (adolescents aged 13-17 years) to explore their interest in VR, views about VR as a way to encourage activity, potential barriers, and which features they would like to see in a VR game. Interviews were conducted by 2 graduate psychology researchers. Data were analyzed using thematic analyses conducted by the academic team (AF, NF, and KN). To incorporate findings into game development, NF presented results as they emerged and created a table of desired game features. Full thematic analyses were performed [46,47] in parallel, fulfilling sufficient sample size requirements [48], and the findings were published (including the table of recommendations provided to the industry partner) in *JMIR Serious Games* in 2019 [49].

A separate qualitative study aimed to understand how parents of adolescents felt about VR as a way to encourage adolescent PA and how best to gain their support for this type of intervention. Summary findings necessary for game development were presented to the industry partners in a meeting, and full findings were published in *JMIR Serious Games* [50].

### Inductive Thematic Analysis of Public Reviews of VR Exergames

To investigate game features that were particularly enjoyed or disliked in VR exergames reported players themselves, we thematically analyzed 498 publicly available reviews of exergames from the top 3 VR marketplaces Steam (Valve Corporation), Viveport (Valve Corporation), and Oculus (Oculus VR). A table of key recommendations was generated for the industry partner as soon as possible to share findings to feed into our game development, and the full study (including the table) has been published in *Journal of Medical Internet Research* [51].

### Survey and Interviews With Zombies, Run! Users

The exergame developed by our industry partner (Zombies, Run) is an immersive audio AR mobile app released in 2012 [52]. The app became the highest-grossing health app on Apple’s App Store 2 weeks post release. Since 2012, Zombies, Run! has accrued 5.5 million downloads, with approximately 200,000 monthly active users [53]. Zombies, Run! combines exercise and a postapocalyptic radio story, a narrative-based game delivered via the smartphone app. To understand what appealed to the users of popular and widely used exergames and their potential impact on PA behavior, we surveyed Zombies, Run! users between November and January 2019. The survey was cross-sectional and included 36 questions around experiences of using Zombies, Run!, likes and dislikes of app features, and engagement with BCTs. Users were also asked to report their PA levels (number of days and length of sessions of MVPA using the items from Coleman et al [54]) before and after using Zombies, Run! The survey also aimed to explore their interest in VR exergaming and perceived benefits and barriers to this. The users were reached via in-app notifications, Zombies, Run! social media, and Zombies, Run! newsletter. The survey asked Zombies, Run! users if they were willing to be interviewed qualitatively to tell us more about their experiences, and 30 Zombies, Run! users were interviewed.

### Building the Prototype

At the conception of the project, the industry partners developed gameplay concepts for the VR prototype based on some key findings that emerged from our research (summarized in Table 3). Six to Start then established some basic principles to widen the potential game audience in terms of technology and appeal:

1. The game should not require any special equipment other than a standard Vive/Oculus VR setup, that is, no weights, additional Vive trackers, exercise bikes, or pull-up bars (because this would limit future implementation).
2. Gameplay should not be overtly about fitness or exercise (because our user group almost universally reported they would prefer a fun game with physical movement as a byproduct).
3. The game must work in a standard household environment, such as a living room with a ceiling with normal height.

Six to Start reviewed a wide set of basic gameplay types, such as Simon Says (similar to Dance Dance Revolution), Dodgeball, Point and Shoot, Building, Plate-spinning (maintenance of a chaotic system, eg, Diner Dash), and Photography. They also established some desirable principles such as quick sessions, peaks and troughs in physical exertion, and quick start-up, all of which are common in very popular *casual* and *hypercasual* games that tend to reach a very wide audience. Finally, both teams discussed the content of the game and how it might be scaled—whether the game’s levels should be all human-authored or randomized or semirandomized. Two broad concepts were arrived at with the working titles *Action Photography* and *Hole in the Wall*. These were discussed with our user group, ultimately to develop solely the latter concept to have more time for iteration and graphical polish. Development took place on the Unity platform and Steam VR to ease cross-platform deployment to different VR hardware. The prototype was designed for room-scale use and tested on HTC Vive hardware with the standard 2 hand controllers to allow for full-body movement tracking. Gameplay was iterated during development, adding music, and developed levels that increased in difficulty and built level design tools for nonprogrammers.

**Table 3:** Key features of research fed into the game design.

Study	Key findings that fed into game design	Reference
Review of the literature	Presented in Table 2	Table 2
User group	Preferences for game types and preferred BCTs^a^ Iterative feedback on prototype ideas	N/A^b^
Adolescent survey	Strong desire to increase PA^c^ Preferred BCTs such as goal setting and feedback on performance	In preparation
Adolescent qualitative interviews	Desired VR^d^ exergame features: being able to exercise at home; rewards; increasing challenges; social or multiplayer aspects; using own music Barriers: high cost; the need for parental buy-in	Published in a study by Farič et al, 2019a [49]
Parent qualitative interviews	Approval of harnessing gaming for something positive Intervention must be nonviolent Mental health most salient reason for encouraging PA, and a calming virtual environment preferred	Published in a study by McMichael et al 2020 [50]
Thematic review of VR exergame reviews	Desired VR exergame features: removing motion sickness owing to the immersive quality and accurate graphics; gradual acquisition of skill and multiplayer options with music Disliked features: motion sickness, poor graphics, and unresponsive developers	Published in a study by Farič et al, 2019b [51]
ZR^e^ survey	Global appeal to those who identified as gamers and nongamers of all ages; primary use of ZR was running but also walking, gardening, cycling, and training for weight and fitness control, even to run marathons	In preparation
ZR interviews	The narrative and interactive storyline as the reason for engagement, not the actual gameplay; a distraction from negative and mundane aspects of running; community feel; more than half used ZR to improve mental health (improved mood)	In preparation

^a^BCT: behavior change technique.

^b^N/A: not applicable.

^c^PA: physical activity.

^d^VR: virtual reality.

^e^ZR: *Zombies, Run!*

### Intellectual Property: Game Code

Establishing intellectual property (IP) agreements with multiple partners can be complex, and establishing an IP contract that satisfies all collaborators took many months. A funder requirement was that, at the end of the project, the code for our game would be made available open source under the Open Source License GPL v3 (General Public License version 3) and available on our website. Open-source code is beneficial for future software development and the academic community. A benefit of acquiring external funding for a research endeavor meant that Six to Start were also involved in an exploratory research capacity, rather than for financial gain. If other partnerships requiring similar IP contracts wish to see the content of the agreement to expedite their own process, they are welcome to contact the corresponding author for sharing.

### Ethical Consideration

The University College London (UCL) Ethics Committee provided ethical approval for all relevant steps described above (Project IDs: 10213/001, 12669/001, and 3777/004) and adolescents or parents provided informed written consent. The results of each stage of this work have been or will be disseminated through presentations at conferences in PA, public health, and gaming, and peer-reviewed publications.

## Results

### Quantitative Survey of Adolescents

A total of 511 adolescents from schools and youth organizations completed the survey. Of these, 48.9% (250/511) were aged 13-15 years, 35.6% (182/511) aged 16-18 years and 15.5% (79/511) were aged 19-24 years. Analyses are ongoing, and we plan to explore differences in preferred BCTs and technology features by gender and age; however, 77.1% (394/511) were interested in advice to increase their PA and most likely to go on the web or use a tablet or mobile app to find information about PA. About 50.0% (256/511) used some kind of health tracker. Preliminary data on preferred BCTs suggest that goal setting, personalized feedback on behavior, instructions on how to perform a behavior, self-monitoring, and rewards were strongly desired. Features that were less desired were information about the health consequences of not performing sufficient PA and features that included social networks or forums or photo feedback.

### Qualitative Interviews With Adolescents and Parents

Qualitative interviews were conducted with 31 adolescents (18/31, 58% female; 19/31, 62% non-White ethnicities). Boys and girls were equally positive about the use of VR for PA promotion. Both highlighted fun or enjoyment as fundamental. Participants identified rewards, increasing challenges, and including social or multiplayer and real-world aspects as important game features. Barriers included the cost of high-end systems and the need for parental approval [49]. Exercising at home was perceived as very appealing and a way to overcome social and cultural barriers to PA, particularly for girls. A total of 18 parents of adolescents took part in interviews and believed that VR exergaming would engage their adolescents with PA, and although they would prefer real-world PA, they were very supportive of an intervention that harnessed gaming for a positive outcome (promotion of PA). In addition, they consistently reported that to garner their support, a game must be nonviolent, and that mental health was their most salient reason for encouraging PA in their adolescents, so a game would ideally be calming or relaxing rather than aggressive. For more details on the results of the studies, see studies by Farič et al [49] and McMichael et al [50].

### Thematic Analysis of Public Reviews of VR Exergames

The results of the review found that VR exergaming was a way to engage with PA in a fun, enjoyable, and playful way, without PA being the focus of the activity. Promisingly, users reported feeling that VR exergaming had provided exertion comparable with *real-world* PA. However, some notable drawbacks were also identified, such as those pertaining to the technology itself (eg, the mechanics of the games and unintuitive controls) and a lack of real-world feeling while playing the games. The full details of this study are available in a study by Farič et al [51].

### Survey of Zombies, Run! Users

This work was in progress at the time of writing, and we plan to explore findings by age and gender, but summary findings are presented here. A total of 6423 participants opened the link, and 83.19% (5343/6423) completed the survey. Participants’ age ranged from 16 to 71 years (mean 33.1, SD 10.1), with 58.62% (3131/5341) of them identified as female, 38.04% (2032/5341) as male, and 2.52% (135/5341) as other. Most participants were White (4498/5341, 84.21%), and approximately half were from the United States (2594/5343, 48.54%). The most common education category was a bachelors’ degree education (1979/5341, 37.05%). Zombies, Run! app usage was associated with a reported mean increase of 84 min in PA per week (95% CI 82-87). Overall, 39.89% (2119/5311) of participants experienced a positive identity shift (from not a runner to a runner). The BCTs or game features with the strongest perceived impact on the PA behavior were positive outcomes of PA, goal setting, and obtaining intrinsic reward (through fun), whereas the least important were obtaining reward money and social comparison. The most popular game features of Zombies, Run! included simulation, customization, self-monitoring, roleplay, and obtaining a within-game reward. A total of 57.92% (3095/5343) had not tried VR exergaming but would like to, 20.11% (1075/5343) had not tried it and did not want to, 7.44% (398/5343) had tried and liked it, 1.59% (85/5343) had tried and did not like it, and 1.94% (104/5343) did not know what it was. By far, the most appealing perceived positive aspects of VR were immersion and fun or enjoyment. The perceived negative aspects were cost and discomfort (heavy or bulky headset).

Preliminary thematic analysis of 30 interviews revealed that people became immersed in PA through the story’s narrative, which motivated them to keep going and distracted them from negative associations with PA. The app was not solely used for running by all participants, but also for walking, gardening, or cycling. A total of 70% (21/30) of the interviewed sample reported positive effects on mental health. The qualitative and quantitative work is being written for publication by NF as part of her PhD work.

### The Prototype

**Figure 1:**
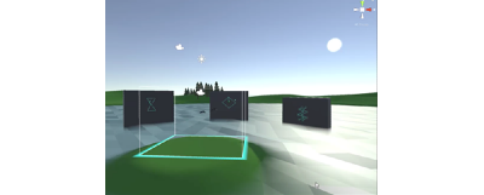
Walls exergame developed and evaluated as part of the project.

**Figure 2:**
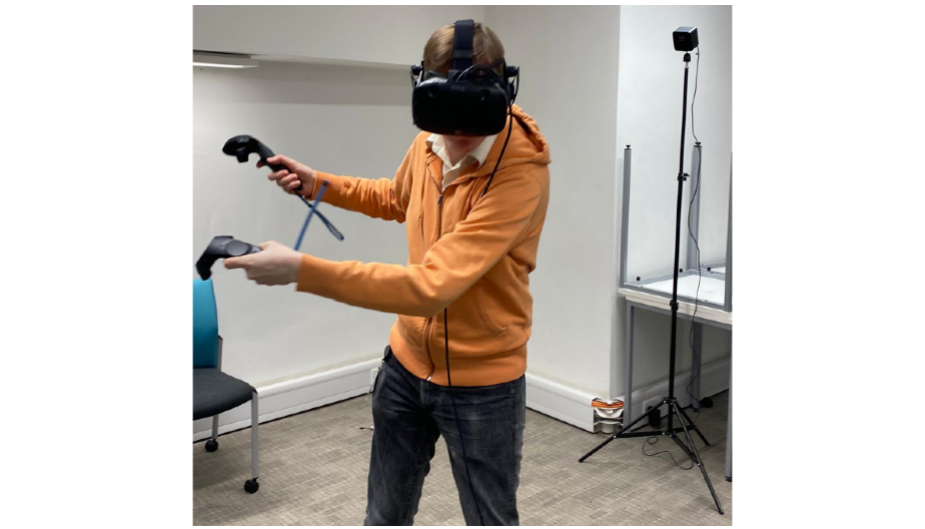
A player during Walls gameplay.

The key findings of the formative research fed into the prototype game are described briefly in Table 3. The game was given the working title *Walls* (although we subsequently asked the user group to suggest appealing names; Figure 1). In the game, the player needs to use the VR controllers to complete complex patterns that appear on walls by moving their arms and body to be as accurate as possible. An image of a player trying the prototype is presented in Figure 2. The accuracy and speed of completion earn players high scores. The game includes PA movements such as stretching, fast and slow arm motions, and dodging. The idea is that if games were developed further, additional levels would become physically challenging. However, the virtual environment was also designed to be calming (based on parental views).

### User Testing of the Prototype

Before trying our prototype, 89% (17/19) of the boys and 59% (10/17) of girls had tried some form of VR. This was almost always a smartphone-based headset rather than a fully immersive experience. All but one who had tried it reported enjoying it, except for one reporting motion sickness. When asked which features they would like to see in our game, goal setting, rewards, social aspects, choosing their own music, custom levels or custom storyline, player creation, and progressive game challenges were reported as the most important features. Most students (but not all) enjoyed the prototype gameplay (23/31, 74%). Issues were lack of instructions at the set up to practice, confusing controller functions, and not seeing the overview of other people’s scores at the end to compare their performance. Almost all students reported feeling excited about the potential of the VR game once it had been further developed and provided additional feedback ranging from alternative names, features that would make the game even more appealing, to ideas for the wider intervention.

### Other Important Outcomes: Training of Researchers and Engaging Adolescents With Science

The formative development work described here has facilitated substantial postgraduate training. In a short funding timeframe, when a large proportion of the grant has to necessarily go toward game development, to conduct and disseminate work, it has been invaluable to involve graduate psychology researchers. In total, 4 UCL Master of Science students (listed in the Acknowledgments sections in this paper and authors on the relevant papers) conducted their dissertations on this work, and the data will contribute to the PhD of NF (who was employed as a research assistant during the funding period). This project’s novelty and the opportunity to spend time in a research center and a gaming lab made the projects appealing and extremely enjoyable and valuable for students. In addition, teachers from the schools where the user groups were recruited informed us that an unanticipated benefit was the engagement of their young people in the scientific process, with a number of their girls and boys expressing feeling motivated to pursue careers in scientific research or computer science or game development.

## Discussion

### Principal Findings

This paper described the formative intervention development work that resulted in the development of a prototype VR exergame designed to engage adolescents with PA. The results of the vEngage formative development phases demonstrate that our target population—younger adolescents aged between 13 and 17 years—see great potential in VR technology as a means of engaging in PA, and this enthusiasm was reflected by users of another exergame population (Zombies, Run!). We identified a number of desired features of VR exergames, including realistic body movements, removing motion sickness and accurate graphics, gradual acquisition of skills, and multiplayer options with music. However, notable perceived drawbacks to a VR exergaming intervention were affordability, accessibility, and potential discomfort. In addition, our study using inductive thematic analysis of public VR exergame reviews suggested that although VR exergaming can elicit levels of exertion that users equate with other forms of MVPA and distract participants from the negative perception of performing PA, the affordability of high-end VR equipment, graphics, and motion sickness are still drawbacks to VR becoming more mainstream [51].

The benefit of the applied design was that academics used traditional academic methods of inquiry to study the target population and, in turn, informed the game developers. Feedback on our prototype was encouraging, and future work (subject to funding) will seek to develop the prototype and wider intervention components further. The academic-industry collaboration was successful because of its iterative nature and frequent meetings where goals were set and the work was performed in parallel and shared in real time. We hope that our process can guide researchers who wish to design an intervention together with their target population and an industry partner. We established ways of distilling top-line academic findings quickly to provide to an industry partner. In addition, we used methods of accessing information quickly, such as analyzing existing publicly available reviews of VR exergames [51] or collecting data from the existing Zombies, Run! user base, along with more traditional recruitment processes.

### Study Strengths and Limitations

This study has several strengths and limitations. The collaborative approach and involvement of end users were strengths. However, within the limits of early-phase funding, it was only possible to develop the game to a standard that would facilitate user input, meaning that we could not assess the game’s potential for impact on PA. We have an ongoing experimental study exploring the potential exertion (heart rate and perceived exertion) that can be achieved by playing some of the popular, commercially available VR games.

The mixed methods were a strength; we employed a number of different methods, including thematic analysis of player reviews, app surveys, standard surveys, and qualitative interviews. However, formative studies had limitations. The quantitative surveys were cross-sectional self-report (although questions about interest and engagement cannot be assessed objectively), and a future aim would be to test the fully developed game and use accelerometers to measure PA. The school-based survey was completed as a class activity following opt-out consent. However, it is very likely that the Zombies, Run! user survey was completed by users who felt most positive about the exergame, introducing some selection bias. Our user group and those interviewed qualitatively were recruited from schools in and around the research center in London, which may not reflect others’ views in different geographical locations (although the sample of interviewed Zombies, Run! users was global). The vast majority of those we surveyed or interviewed did not own or use VR frequently, which meant that barriers and benefits were generally perceived rather than based on experience. Prolonged and frequent exposure in the context of a trial would allow us to explore how these factors influence their PA behavior and user experience. A multimodal intervention would likely be required, targeting multiple influences on adolescent PA [17,18], and this work only focused on VR exergaming (which we hypothesized may enhance motivation for PA). In line with frameworks such as the COM-B model (capability, opportunity, motivation, behavior) and behavior change wheel, it is important that an intervention considers the broader influences on behavior and not just individual motivation [42].

### Long-Term, Sustained Behavior Change

It is worth considering the broader application of our findings, including steps toward implementation of VR (and AR) exergaming interventions. Implementation of VR games to sustain behavior change would be made easier if VR gyms or fitness centers using VR existed in the United Kingdom, but at present (August 2020), there are none, and the cost of an average head-mounted VR is still relatively high (average Can $585 [US $460]). The virtual gyms (eg, Digme) that do exist do not allow full immersion via head-mounted pieces but modify the user’s experience by similar means such as theater performances and AR (eg, use of music and lights) and display certain features on the screens in front of users [55]. The availability and accessibility of VR for PA is still tied to cost, the technological market, and PA competitors (eg, gyms, fitness clubs, sports centers, apps, and outdoors). VR exergaming would have to offer people a distinct and accessible PA experience. However, it has been predicted that VR console homeownership will continue to rise [32]. The COVID-19 pandemic and the need for social distancing or restrictions placed on exercise facilities mean that tools that can facilitate home-based exercise have never been more important.

### Future Work

There are a number of challenges in the next steps of the project. First, funding must be acquired for future phases. The funding was awarded for formative development work, and we believe that we have established that VR exergaming is promising for engagement with PA in our target group. However, significant development work is required for the game to be at the stage where it is ready to trial. As highlighted in the introduction, a multimodal intervention would likely be required to facilitate sustained PA change [17,18]. We have always acknowledged that the game alone is unlikely to be sufficient for long-term change and working with our user group identified that linking with *real-world* technologies (eg, trackers) and real-world activity partners (eg, vouchers to try a range of real-world activities like climbing wall, boxing class, and trampoline park). However, these options will have to consider the post–COVID-19 pandemic world and the need for socially distanced exercise, which our vEngage project did not directly address. The implementation also requires the game to be promoted in order to gain a sufficient user base to support further testing and warrant further development. To be successful as more than a testbed, the game needs a funding mechanism beyond the initial research funding.

Future intervention development steps (phase II) include developing the VR game to a high specification, identifying components that can link PA to the game (eg, smartphone phone tracking), linking with real-world PA partners (eg, schools and community or leisure centers, gaming conferences), and linking with vloggers (video bloggers). Ultimately, we would like to empirically test our intervention’s potential to enhance motivation and change behavior (phase II).

### Conclusions

This vEngage project is the first attempt to develop a VR exergame designed to engage adolescents with PA using an academic-industry collaboration [56]. Our findings suggest that VR exergaming has potential as a public health intervention designed to engage adolescents in PA. We plan to take this work forward and invite collaboration for future stages.

## Abbreviations

AR: augmented reality
BCT: behavior change technique
HIT: high-intensity training
IP: intellectual property
MRC: Medical Research Council
MVPA: moderate-to-vigorous physical activity
PA: physical activity
SDT: Self-Determination Theory
UCL: University College London
VR: virtual reality
